# Corn Stunt Disease: An Ideal Insect–Microbial–Plant Pathosystem for Comprehensive Studies of Vector-Borne Plant Diseases of Corn

**DOI:** 10.3390/plants9060747

**Published:** 2020-06-14

**Authors:** Tara-kay L. Jones, Raul F. Medina

**Affiliations:** 1 Department of Entomology, Texas A&M University, TAMU 2475, College Station, TX 77843-2475, USA; jonestarakay1@tamu.edu; 2 Texas A&M AgriLife Research—Weslaco, 2415 E. Business 83, Weslaco, TX 78596-8344, USA

**Keywords:** vector-borne, phytopathogens, insect vectors, corn stunt

## Abstract

Over 700 plant diseases identified as vector-borne negatively impact plant health and food security globally. The pest control of vector-borne diseases in agricultural settings is in urgent need of more effective tools. Ongoing research in genetics, molecular biology, physiology, and vector behavior has begun to unravel new insights into the transmission of phytopathogens by their insect vectors. However, the intricate mechanisms involved in phytopathogen transmission for certain pathosystems warrant further investigation. In this review, we propose the corn stunt pathosystem (*Zea mays*–*Spiroplasma kunkelii*–*Dalbulus maidis*) as an ideal model for dissecting the molecular determinants and mechanisms underpinning the persistent transmission of a mollicute by its specialist insect vector to an economically important monocotyledonous crop. Corn stunt is the most important disease of corn in the Americas and the Caribbean, where it causes the severe stunting of corn plants and can result in up to 100% yield loss. A comprehensive study of the corn stunt disease system will pave the way for the discovery of novel molecular targets for genetic pest control targeting either the insect vector or the phytopathogen.

## 1. Introduction

More than 700 distinct plant diseases identified as vector-borne pose a significant threat to plant health and food security within agricultural systems all over the world [1,2,3,4]. The study of interactions between insect vectors, phytopathogens, and their shared plant hosts is fundamental for the elucidation of clues necessary for developing novel and sustainable control strategies to mitigate losses caused by vector-borne plant diseases. When it comes to understanding the molecular mechanisms involved in insect–pathogen and vector–pathogen interactions, the majority of what we know comes from the studies of *Drosophila* [5,6] and *Aedes* or *Anopheles* mosquitoes [7,8,9]. While studies using model organisms such as mosquitoes have been important for improving our understanding of pathogen infection mechanisms, this model pathosystem only provides information about the transmission of human and animal diseases. Thus, there is a need to increase our knowledge of the mechanisms used by vectors of plant diseases. In recent years, a surge of studies focusing on hemipteran insect vectors has emerged [10]. However, most of the plant pathogens being studied are plant viruses transmitted in a non-persistent or semi-persistent manner (e.g., *Alfamovirus*, *Fabavirus*, *Cucumovirus*, *Potyvirus*, *Closterovirus*, *Sequivirus*, and *Crinivirus*) [11,12,13,14,15,16]. There are also comparatively fewer studies on vector transmitting bacteria, particularly of class Mollicutes than on vector-transmitted viruses. Insect vectors of non-persistently and semi-persistently transmitted viruses only remain viruliferous for a short period (seconds to minutes or hours to days but not beyond the molting process of their vectors) [17], and thus a single individual has low chances of transmitting a pathogen to several plants within a field. Devising novel strategies for controlling rapidly spreading diseases in plants will require an increase in the type of phytopathogens and vectors studied. For example, phytopathogens that are persistently transmitted by insects remain within their vectors for prolonged periods (several weeks to months), making them of great significance in the long-term dissemination and widespread movement of vector-borne diseases that are typically harder to control using traditional strategies. Moreover, the majority of studies detailing persistent transmission have focused on phytopathogens that infect dicotyledonous plants such as tomatoes, beans, and peppers [18]. In this review, we call for additional and more extensive studies to fill the knowledge gap that exists in our understanding of the spread of vector-borne phytopathogens that are persistently transmitted to monocotyledonous plants. We propose the use of corn stunt disease, which is caused by *Spiroplasma kunkelii* and that is persistently transmitted by *Dalbulus maidis* to *Zea mays,* as an ideal model system for dissecting the molecular determinants and mechanisms underpinning the insect vector–mollicute interaction of an economically important monocotyledonous crop.

Corn stunt disease has been the most important limiting factor of corn production in the Caribbean and the Americas, including the United States, Mexico, and Central and South America during the 19th and 20th centuries [19,20,21,22,23]. Recently, corn stunt disease has resurfaced, as symptoms were observed in native corn varieties grown in southeast Puebla, Mexico [24]. Corn stunt disease results in severely stunted plants that often produce multiple small ears with loose and/or missing kernels. This disease is caused by *Spiroplasma kunkelii*, commonly known as the corn stunt spiroplasma (CSS). This bacterial pathogen is transmitted singly or in combination with *Maize bushy stunt phytoplasma* (MBSP) and/or *Maize rayado fino virus* (MRFV) to healthy corn plants by the corn leafhopper, *Dalbulus maidis*, in a persistent-propagative manner. *Dalbulus maidis* is considered a serious pest of corn in Mexico, the Caribbean basin, and Central and South America, especially because of its competence in phytopathogen transmission and associated yield loss [25,26,27]. For example, Brazil’s corn production during the 2017–2018 period was impacted by a severe *D. maidis* infestation [28]. Efforts to control *D. maidis* in Brazil led to economic constraints to local corn producers due to an 85% increase in pesticide usage compared to the previous seasons. There is no current control strategy established for managing corn stunt disease by directly targeting this phytopathogen. The use of insect-resistant corn germplasm has thus far been unsatisfactory [29]. Control methods, therefore, rely on attempts to either suppress or completely eradicate the insect vector, thus resulting in indirect pathogen control [27]. Indirect corn stunt disease control has been achieved through the application of insecticides, but it is debatable as to whether it produces a significant net return [30]. In addition, the application of insecticides has been recorded as having a potential negative impact on parasitoids and predators of *D. maidis* [30].

In nature, spiroplasmas exist as both pathogenic (e.g., *S. kunkelii* causing corn stunt disease in corn, *S. citri* causing citrus stubborn disease in several plant families, and *S. phoeniceum* causing the periwinkle yellows disease in periwinkle plants (Table 1)) and non-pathogenic microbes (e.g., *Spiroplasma* spp. strains in *Drosophila* co-exist as symbionts) [31]. Pathogenic spiroplasmas have evolved a diverse host range that extends from plants, insects, and crustaceans to most recently affecting humans directly (e.g., a human-infecting spiroplasma was newly identified as a strain of the horse fly symbiont, *Spiroplasma turonicum* [32]). Arthropod vectors play an important role in the spread of plant pathogenic spiroplasmas, and thus it is important to understand how they interact with their vectors and cause disease epidemics in agricultural settings. In total, mollicutes (i.e., spiroplasmas and phytoplasmas) are responsible for causing plant diseases in over 300 crops [33]. Hence, it is of significance to understand the specific associations and mechanisms that regulate the spread of these phytopathogens in agricultural crops.

Many insect vector–phytopathogen–plant interaction studies focus on the vectoring capacity of insect species that threaten (through a combination of herbivory and pathogen transmission) a wide host plant range, as this is necessary to justify a significant economic threat. Therefore, generalist phytophagous insects, which can pose a serious threat to several different plant species from distinct families, are often the preferred choice of study. On the other hand, specialist phytophagous insects that demonstrate feeding behaviors highly specialized on a single host plant species or just a few related ones are lagging [34]. It is also important to recognize that insect vectors, phytopathogens, and host plants form successful interactions through specialization [35]. For example, insect–host plant specificity generally offers increasing developmental benefits in terms of biochemical compatibilities, thus boosting the survivability of the insect species to tolerate plant defenses, manipulate host plants to their benefit, and evolve ways to reduce predation and parasitism [36,37,38,39]. Understanding specialized interactions between insect vectors and phytopathogens with their shared host plants can help us piece together the mechanisms and strategies that are used to facilitate herbivory and vector-borne disease epidemiology. Thus, the corn stunt pathosystem could be exploited as a model system that demonstrates successful interactions through specialization (i.e., *D. maidis* has coevolved and developed as a specialist of corn as an efficient vector of *S. kunkelii*) [40,41,42,43,44], which makes it ideal for understanding the intricate interactions of phytopathogens persistently transmitted by insect vectors.

Over the last two decades, research on genetics, molecular biology, physiology, and behavioral studies has brought new insights into the mechanisms involved in the insect transmission of phytopathogens. Discoveries from these disciplines have been revolutionized by the development of genetic technologies, including RNA interference (RNAi) and Clustered Regularly Interspaced Short Palindromic Repeats (CRISPR) [45,46,47]. These new genetic tools offer a promising future for agriculture through genetic pest control, including the design and implementation of novel strategies for managing vector-borne plant diseases. Genetic pest control employs molecular tools to manipulate pest genes, with the primary aim of reducing their population densities [48]. These genetic tools can be further exploited to abolish pathogen transmission without reducing or eliminating an insect vector from a given geographical area. A number of insect pests of crops, as well as human disease vectors, are currently undergoing laboratory assessments and open-release trials [49,50]. For example, the population suppression or replacement of laboratory *Aedes* and *Anopheles* mosquitoes that are vectors of arboviruses and malaria, respectively, has been achieved using genetic pest control strategies that utilize a gene drive mechanism (reviewed in Macias et al. [51]). Similarly, these may be applied to protecting agricultural systems by specifically targeting and disrupting pathogen transmission by rendering vectors refractory to infection or just abolishing pathogen acquisition and/or transmission [51,52,53,54,55,56]. To find successful candidates for genetic pest control, much of the recent studies have focused on first achieving a more comprehensive understanding of the complex series of events leading to successful insect vector-phytopathogen-plant interactions [10]. In our proposed model, the corn stunt pathosystem is expected to contribute to the discovery of key candidate genes that could be useful targets for genetic pest control of an insect vector and plant-infecting spiroplasma threatening corn, a staple crop of worldwide importance.

**Table 1:** List of plant diseases caused by *Spiroplasma* pathogens, their leafhopper vectors and host plant families.

Plant Disease	Spiroplasma Pathogens	Leafhopper Vectors	Host Plant Families	References
Citrus stubborn disease	*Spiroplasma citri*	*Circulifer haematoceps, Scaphytopius nitridus Circulifer tenellus,* *^E^ Circulifer opacipennis,* *^E^ Macrosteles fascifrons* Fieber, *Ricaria japonica*	Alliaceae, Apiacceae, Apocynaceae, Amaryllidaceae, Asteraceae, Brassicaceae, Brassicaceae, Crassulaceae, Cucurbitaceae, Plantaginaceae, Rosaceae, Rutaceae, Scrophulariaceae, Violaceae	[57,58,59,60,61,62,63,64,65,66]
Corn stunt disease	*Spiroplasma kunkelii*	*Dalbulus maidis, Dalbulus elimatus,* *Dalbulus guevari,* *^E^ Dalbulus gelbus* *^E^ Dalbulus quinquenotatus* *^E^ Dalbulus tripsacoides* *^E^ Dalbulus longulus* *^E^ Exitianus exitiosus,* *^E^ Euscelidius variegatus* *^E^ Graminella nigrifrons,* *^E^ Stirellus bicolor,*	Poaceae	[21,25,67,68,69]
Periwinkle yellows disease	*Spiroplasma phoeniceum, Spiroplasma citri*	*^E^ Macrosteles fascifrons*, *^E^ Cicadulina bipunctella* *^P^ Phlogotettix Cyclops* *^P^ Balclutha* sp.	Apocynaceae	[56,59,70,71]

^E^ Experimental insect vectors, ^P^ potential insect vectors (insects tested positive for *Spiroplasma* in the field but with insufficient evidence of transmission).

## 2. Components of Corn Stunt Disease

### 2.1. Dalbulus Maidis

**Figure 1:**
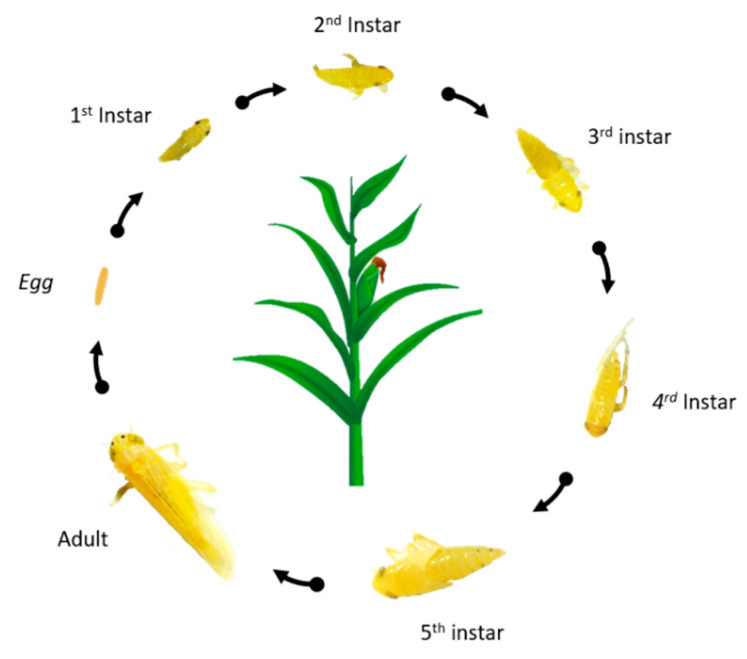
The complete lifecycle of *Dalbulus maidis*. *D. maidis* begins as an egg and then undergoes five nymphal instars before reaching adulthood. It takes around 4 to 6 days from oviposition to the emergence of the first nymphal instar. The optimal developmental range for each nymphal stage averages from 3 to 4 days. Adult longevity varies among males and females from an average of 78 and 30 days, respectively. Mature females oviposit an average of 15 eggs per day for most of their adult life [78]. Photo credit: Tara-Kay L. Jones.

The corn leafhopper, *Dalbulus maidis* (DeLong and Wolcott) (*Hemiptera*: *Cicadellidae*), is a phloem-feeding specialist of the genus *Zea,* which evolved in an increasingly close association with modern-day maize cultivars [40,72,73]. *Dalbulus maidis* undergo a hemimetabolous lifecycle (Figure 1) and only reproduces on species of *Zea*. Although *D. maidis* only reproduce on species of *Zea*, it is known to feed on several other monocotyledonous plant species such as gamagrass and Johnson grass in the absence of corn and teosintes [74]. There is evidence of *D. maidis* surviving for several months in the absence of host plants in non-irrigated maize-free fields [75]. Its distribution range extends throughout most of the American continent and the Caribbean islands, with Mexico being reported as the center of origin [76]. Under optimal environmental conditions, *D. maidis* can colonize corn fields quickly and cause severe yield losses, thus resulting in drastic economic losses [77]. For example, during the period 1979–1980 *D. maidis* occurred in south Florida, United States, and caused a 98.5% decline in the state’s expected corn yield due to direct feeding as well as through disease spread [26,78], which translated into $60 million lost in corn production [20,79].

### 2.2. Spiroplasma kunkelii

*Spiroplasma kunkelii* is a characteristically helical motile bacterium from the class Mollicutes. These cell wall-less bacteria have an internal cytoskeleton, which is composed of fibrils located under the cell membrane [63]. *Dalbulus maidis, D. elimatus*, and *D. guevari* are natural vectors of *S. kunkelii* but there are other leafhopper species (*D. gelbus, D. quinquenotatus, D. tripsacoides, D. longulus, Exitianus exitiosus, Graminella nigrifrons, Stirellus bicolor*, and *Euscelidius variegatus*) that have been experimentally proven to transmit the pathogen to healthy corn plants under laboratory conditions [21,25]. The *Spiroplasma kunkelii* (CR2-3X strain) genome has been fully sequenced and consists of a 1,463,926-bp circular chromosome and four plasmids [68,80,81]. Within the *S. kunkelii* chromosome, there are 1646 protein-coding regions, multiple insertions of *Spiroplasma* virus sequences, a set of rRNA genes, and 33 tRNA genes [68]. The availability of *S. kunkelii* genomic information facilitates studies of pathogenicity and evolutionary adaptations with plants and insect vectors, respectively, which makes it a suitable candidate for studying its interaction within these disparate hosts where it replicates but causes very distinct outcomes. In addition, the availability of *S. kunkelii* genomic data is also useful for conducting studies aimed at better understanding the mollicute components that regulate transmission by its insect vectors. Phylogenetically, *S. kunkelii* clusters with other bacteria in the class Mollicutes and it is most closely related to the plant pathogens *S. citri* and *S. phoeniceum*, which affects citrus and periwinkle, respectively [82,83,84]. Additionally, *S. kunkelii* is also in the same genetic cluster as the honeybee pathogen, *S. melliferum*, and thus it could potentially provide insights on the pathogenicity of these distinct organisms [82,83]. While an increasing amount of literature is being published on *S. citri* due to the economic and agricultural importance of citrus crops, it is important to note that *S. citri* has been reported to have a pathogenic effect on its insect vector as opposed to *S. kunkelii* which seemingly forms an either positive or neutral association with *D. maidis*, as it has not been found to have a detrimental effect on the insect’s longevity or fecundity [85,86]. Therefore, more comprehensive studies on the interactions of *S. kunkelii* and *D. maidis* are likely to increase our understanding of the molecular bases and biological consequences resulting in the efficient transmission of this mollicute by its main leafhopper vector to maize.

### 2.3. Zea mays

*Zea mays*, commonly known as corn or maize, is a member of the grass family *Poaceae*. Corn is a diploid, large grain cereal that originated in Mexico and disseminated further north and south of its center of origin. Corn is one of the most important cereal crops worldwide, ranking third in production following wheat and rice. Furthermore, it has a diverse distribution range, with its capacity to grow on all continents except Antarctica [87]. Corn plays a significant role in human nutrition (it contains 60–68% starch, 7–15% protein, and is rich in amino acids and minerals such as phosphorus and potassium), it is extensively used for animal feed and is also utilized for biofuel production as well as in other numerous industrial products [88]. Corn has been established as a genetic model monocotyledonous plant for over a century [89], which makes it an ideal choice for studying molecular interactions amongst insect vectors and their associated phytopathogens in a monocotyledonous crop. There are seven known vector-borne diseases of corn, five of which are viral (i.e., *Maize dwarf mosaic potyvirus* (A and B), *Maize stripe tenuivirus*, *Maize rayado fino marafivirus*, and *Maize mosaic nucleorhabdovirus*) while the other two are bacterial (i.e., corn stunt spiroplasma and maize bushy stunt phytoplasma). Interestingly, three of the seven vector-borne diseases affecting corn’s production are caused by phytopathogens transmitted by *D. maidis* in a persistent-propagative manner. Corn pathogens transmitted by *D. maidis* have been reported in all corn-growing regions where the insect is present and have caused damage, ranging from 40%–100% yield loss. Limited scientific information is available regarding the targets and mechanisms of resistance in corn against Spiroplasma infections [67]. For this reason, it is important to understand the components of the corn stunt disease. Studies focusing on the mechanisms of corn pathogen transmission could provide us with major clues on the interactions within this insect vector–phytopathogen–plant triad that might aid in disrupting transmission and offering sustainable control strategies against plant diseases in this and/or other monocotyledonous plants.

## 3. Insect Vector Interactions with Host Plant and Pathogen

### 3.1. Dalbulus maidis–Spiroplasma kunkelii

The transmission route of *S. kunkelii* in its *D. maidis* vector has been documented by several researchers [33,90,91]. *Spiroplasma kunkelii* is acquired by *D. maidis* as early as several minutes of the insect feeding from the sieve tube of the phloem of infected corn plants. The pathogen is then en-route to the gut, where it is mixed with the gut fluids and then crosses the midgut barriers to enter the hemocoel where it primarily replicates in the hemolymph and follows a complete systematic circulation [33]. The pathogen has a latent period that ranges from 18 to 22 days (depending upon the isolate and titer of the pathogen, as well as depending upon the biotype and age of the insect vector) before it can enter the salivary glands to finally be transmitted to healthy plants. Within the insect vector, *S. kunkelii* localizes in tissues and organs such as the ovaries, fat body, cytoplasmic vesicles, and salivary glands, and it is also detected in the gut lumen, where it is retained within a bacterialiferous vector for up to 45 days [33,90].

The leafhopper vector transmission of *Spiroplasma* is mediated by hemolymph proteins, functional peptides, pathogen proteins, and enzymes. To date, there has not been any extensive study of functional vector derived proteins and pathogen proteins involved in the *D.maidis* transmission of *S. kunkelii*. In a related Spiroplasma species, *S. citri*, a correlation exists between the leafhopper transmission efficiency and *S. citri’s* ability to bind a phosphoglycerate kinase (PGK) to the leafhopper vector’s actin [92]. This binding of *S. citri* PGK to its leafhopper vector’s actin protein resulted in the cellular internalization of the phytopathogen [92]. Leafhopper membrane proteins have been hypothesized to play important roles in the spiroplasma barrier crossing and transmission through the process of receptor-mediated endocytosis [33,92,93,94]. Some potential transmission proteins already identified are solute-binding proteins (e.g., Sc76 and P32 protein identified in *S. citri*); adhesion-associated proteins (e.g., ScARPs); and spiralin, which has been shown to bind with leafhopper’s glycoproteins [92,95]. The genome sequence of *S.kunkelii* revealed the presence of putative virulence genes encoding P123, P58, P54, and P18 which are also present in the transmissible *S. citri* genome and represent a deletion in the non-transmissible *S. citri* lines [80]. Functional molecular analyses conducted by Breton et al. [96] identified the role of pScil-6 plasmid in conferring the transmissibility of *S. citri*. In this study, Breton et al. [96] further elucidated that the protein pE (a derivative *S. citri* plasmid) was a replicon protein necessary for the replication of pScil-6. A *soj*-encoded polypeptide was also found to be likely involved in plasmid partitioning, and together these proteins enable the transmissibility of Spiroplasma [96]. While these proteins have been identified in a closely related spiroplasma-insect vector pathosystem, further analysis is necessary to uncover more candidates and further elucidate their functional role in the transmission cycle of *S. kunkelii* by *D. maidis*.

Comparatively, non-pathogenic Spiroplasmas, such as those detected in *Drosophila* species, exhibit strategies to evade host immune defenses and result in commensalism interactions that accommodate their survival and transmission at no fitness cost to the host [97,98]. In contrast, other non-pathogenic spiroplasmas have a more mutualistic interaction with their insect hosts, such in *Acyrthosiphon pisum*, where fitness costs associated with hosting spiroplasmas are compensated by increased defenses against threats such as parasitism [99]. Additionally, in *D. melanogaster,* the presence of non-pathogenic Spiroplasmas may co-occur with *Wolbachia* symbionts, which have been found to reduce the pathogenicity of infectious *Photorhabdus luminescens* bacteria as well as triggering immune-signaling responses [97]. Non-pathogenic Spiroplasmas are generally restricted to the gut of their insect hosts compared to pathogenic Spiroplasmas, which can invade the hemolymph and other host tissues [90]. Another differentiating trait related to the pathogenicity of Spiroplasmas are sex-ratio disorders, which have been observed in pathogenic strains [100]. For example, *S. poulsonii* in tropical Drosophila species, during transovarial transmission, kill male progeny of infected females [101]. In some beetle species, Spiroplasma male-killing has been documented [102,103]. There is no published evidence of male-killing in insect vectors of plant diseases. The exploration of *D. maidis*-*S. kunkelii* interactions could reveal vector fitness costs and open a path for us to hijack these mechanisms as part of genetic vector control strategies.

### 3.2. Dalbulus maidis–Zea mays

The corn leafhopper interaction with corn is thought to be mediated by evolutionary mechanisms triggered throughout corn’s domestication from its teosinte relative, the annual teosinte *Zea mays* subsp. parviglumis Iltis and Doebley, over 9000 years ago in central Mexico [42,104]. To date, little is known about the genetic mechanisms mediating *D. maidis* successful adaptation to corn, except that there are *D. maidis* populations that are genetically distinct from each other across the transition from wild to domesticated corn and well adapted to either one of these different host plants [42]. Several studies that previously assessed the *D. maidis* and corn interaction have specifically looked at the insect vector performance (e.g., survivorship, and reproduction) [26,40,73,105,106]. For example, Moya-Raygoza and Garcia-Medina [106] showed that the reproductive capacity of *D. maidis* plays a crucial role in its colonization success. The higher reproductive capacity of *D. maidis* has been proven to be a positively correlated factor of higher population density in regions of Argentina and Mexico [106]. On the other hand, the plant chemistry of corn (particularly the expression of jasmonic acid (JA) and salicylic acid (SA)) could explain at least in part the defense mechanisms that might play a role in the adaptation and performance of *D. maidis* on corn compared to teosintes [107,108]. For example, *D. maidis* herbivory has been shown to cause a higher expression of SA on corn, while this same challenge induced a higher expression of JA in teosintes, suggesting that this defense response has changed throughout corn breeding and domestication [109]. These findings provide a reasonable clue to potential evolutionary patterns in *D. maidis* adaptation that might dictate the differences observed of higher abundance of this insect vector in corn than in its teosinte progenitor [110]. Although not yet tested, there might also be other genetic components contributing to the successful performance of *D. maidis* on corn compared to teosintes. Therefore, we also proposed more comprehensive molecular and genetic studies to further elucidate the interaction between *D. maidis* and corn that ultimately benefits pathogen acquisition and transmission from its confinement within the plant host vasculature.

## 4. Plant–Pathogen Interaction

### 4.1. Zea mays–Spiroplasma kunkelii

*Spiroplasma kunkelii* colonizes and moves through the flowing photosynthate (sugars and other energy-rich compounds) within the phloem of corn plants and localizes in the actively growing regions such as the younger leaves, roots, flowers, and fruits [19]. The symptoms of corn stunt disease vary according to the titer of *S. kunkelii* in infected corn plants and the susceptibility of the host plant [111]. Typically, diseased corn plants are identified by chlorotic stripes, which extend from the base of the leaf laminas towards the apex or leaf margins, red to purple streaks, as well as stunted growth as a result of the shortening of internodes [111,112]. Similar corn stunting symptoms are caused by *Maize bushy stunt phytoplasma* (MBSP), where corn plants are observed with small reddish leaves and/or leaves with red midribs or leaves with yellow or chlorotic stripes. However, disease diagnosis can be achieved by the detection of *S. kunkelii* and/or MBSP from cornfield samples using laboratory techniques such as enzyme-linked immunosorbent assay (ELISA) [113] and polymerase chain reaction (PCR) [114,115]. The symptom expression of corn stunt disease ranges from 15 to 40 days of initial transmission of *S. kunkelii* by *D. maidis* [20,111]. In severe corn stunt disease, there are generally reduced to no corn kernels produced.

### 4.2. Plant Immunity

Plant defense mechanisms are triggered by the presence of infectious agents and/or stimulated by the infestation of an herbivore [116,117,118,119]. Upon contact with plant tissues, pathogens and herbivores release molecules which are known as elicitors [120,121,122,123,124,125]. Elicitors are categorized as either general or specific. General elicitors are associated with chemicals, herbivores, pathogens, and non-pathogenic/or beneficial microbes. General elicitors of insects are molecules designated as DAMPs (Damage-associated Molecular Patterns), whereas those of pathogens are PAMPs (Pathogen-Associated Molecular Patterns) [126,127]. DAMPs and PAMPs are not specific to host plant cultivar and trigger general resistance. The recognition of DAMPs and PAMPs mediated by plasma membrane-localized pattern-recognition receptors (PRRs) triggers a cascade of reactions within the host plant that leads to a primary innate immunity (basal resistance) called PAMP-triggered immunity (PTI) [128]. Specific elicitors are unique molecules, referred to as avirulence (Avr) proteins and are formed by specialized pathogen races or strains that induce resistance responses that are related to specific host plant cultivars that carry a complementary disease resistance gene (*R* gene) [129,130]. The recognition of Avr proteins and the expression of the complimentary *R* gene by host plants leads to a secondary innate immunity called effector-triggered immunity (ETI) [131]. The activation of both PTI and ETI reduces infection within a host plant [132,133,134]. Because corn is an established model crop, there are already identified cellular DAMPs and PAMPs response paths (for example, the early production of reactive oxygen species (ROS) [135,136], the activation of mitogen-activated protein kinases (MAPK) [137,138,139], and Ca2+ signaling [140]), as well as pathogen recognition receptors (PRRs) (for example, receptor-like kinases (RLK) and receptor-like protein (RLP)) that are activated and are known to have roles in pathogen immunity and herbivory through elicitor-binding [141,142,143,144]. Though a detailed framework on corn defense strategies is already known, further studies are needed to provide information on how pathogens such as *S. kunkelii* and insect herbivores such as *D. maidis* evade and manipulate the complete expression of corn resistance. For example, a study of plant-pathogen molecular interaction by Oliveira-Garcia and Deising [145] shows that a down-regulation of key effector components (i.e., β-1,6-glucan synthesis genes *KRE5* and *KRE6*) in the biotrophic hyphae of *Colletotrichum graminicola* manipulate the expression of PTI in corn and facilitate the compatibility necessary for infection to occur. Similarly, Levy et al. [146] studied the molecular interaction between *Candidatus* Liberibacter solanacearum and tomato plants. In the said study, Levy et al. [146] identified an important protein effector (Lso-HPE1) which plays a role in the suppression of plant immune response. The identification of key effector proteins that suppress plant defense response and thus promote disease susceptibility in plant can be used as targets in developing genetic control strategies against plant disease agents. Similar studies as Oliveira-Garcia and Deising [145] and Levy et al. [146] can be conducted using the corn stunt pathosystem to unravel important details involved in the defense and resistance of bacterial diseases that are plaguing monocotyledonous crops.

## 5. Conclusions and Future Perspectives

The corn stunt pathosystem (*Zea mays*–*Spiroplasma kunkelii*–*Dalbulus maidis*) is identified as a suitable model system for increasing our understanding of vector-borne plant diseases that pose a threat to global food security. Although significant research efforts are aimed at increasing our understanding of vector-borne diseases, there is still limited scientific knowledge of the molecular mechanisms mediating specific vector-borne diseases. To fill this gap in knowledge and be able to devise novel control strategies against vector-borne plant diseases, it is essential to accumulate information involving specialist vectors and their role in bacterial pathogen transmission and disease development in monocotyledonous plants. The mechanisms mediating bacterial disease development in monocots and the transmission of pathogens by specialist insect vectors is an understudied area of insect–pathogen–plant interaction [147,148]. Therefore, in this review we encourage experimental research aimed at improving our understanding of how specialized biological systems (e.g., specialist vectors transmitting plant bacterial pathogens) interact. The interaction between *Dalbulus maidis*, *Spiroplasma kunkelii,* and *Zea mays* meets the criteria of a specialized biological system, as we have discussed throughout this review. Using the corn stunt pathosystem to study the mechanisms of vector-borne disease can contribute to our understanding not only of Spiroplasma-related plant diseases but also of phytoplasma and viral plant diseases, as *D. maidis* can be described as a super-vector due to its efficiency in transmitting MBSP (a phytoplasma pathogen) and MRFV (a viral pathogen) to corn plants. Scientific insights provided by the study of the corn stunt pathosystem may be applicable to other pathosystems (e.g., Bermudagrass stunt disease and Ratoon stunt disease of sugarcane, which cause major challenges in several other plants [149,150,151]). Finally, studying the molecular interactions taking place during corn stunt disease infection may provide candidate genes and molecules that can be targeted as part of genetic pest control strategies that can be used for the successful disruption of pathogen transmission by insect vectors to host plants.
